# Fathers' mental Ill-health and child maltreatment: A systematic review of the literature

**DOI:** 10.1016/j.childyouth.2023.107317

**Published:** 2024-02

**Authors:** Ian Holdroyd, Paul Bywaters, Robbie Duschinsky, Taurean Drayak, John Taylor, Barry Coughlan

**Affiliations:** a Department of Public Health and Primary Care, University of Cambridge, UK; b Human and Health Sciences, University of Huddersfield, UK; c Independent Scholar

**Keywords:** Paternal, Mental health, Maltreatment, Abuse, Neglect

## Abstract

**Background:**

Parental mental ill-health is often described as a risk factor for child maltreatment. Yet the literature commonly foregrounds maternal mental ill-health. To obtain a more complete picture, it is crucial to also understand the associations between fathers’ mental health and child maltreatment.

**Aim:**

To provide a narrative synthesis of evidence about the relationship between fathers’ mental health and child maltreatment.

**Method:**

Four electronic databases were searched, identifying 5479 citations. 151 studies were brought to full-text review. 37 were included in the study.

**Results:**

Studies revealed mixed evidence for associations between forms of paternal mental ill health and child maltreatment, with stronger evidence for paternal depression and weak or no evidence for PTSD and anxiety. Many confounding factors were identified across the papers.

**Discussion:**

The small number and limited range of good quality studies indicate the need to correct the relative invisibility of fathers within research about mental health and child maltreatment. At present, the available evidence is not sufficient to draw firm conclusions about the association between fathers’ mental health and child maltreatment or appropriate policy and practice responses.

## Highlights

• A small and limited number of good quality studies have focused on the association between fathers’ mental health and child maltreatment.
• Of the few studies on this topic, most have focused on common mood disorders such as depression and anxiety.
• There is some evidence suggest that there’s a significant association between depression and physical abuse, yet the magnitude of this association remains unclear.
• Overall the available evidence is not sufficient to make strong conclusions about the association between fathers’ mental health and child maltreatment.

## 1 Introduction

Parental mental health difficulties are often described as an important risk factor for child maltreatment (CM). Early evidence for this association comes from a review of the applied literature in which psychological characteristics such as impulse control were conceptualised as one of the main predictors of CM (Spinetta & Rigler, 1972). Child welfare research in the 1980s tended to provide some support for this hypothesis (e.g., Oates et al., 1985, Zuravin, 1989), identifying statistically significant associations between parental mental ill health and CM. Over the past three decades, various studies (Doidge et al., 2017, Berger, 2005) and reviews (Hindley et al., 2006, Ayers et al., 2019) continued to identify associations between parental mental health difficulties and CM, along with other factors including socioeconomic wellbeing, intergenerational abuse and personality traits (van IJzendoorn et al., 2020).

Data from applied contexts provides further support for the relationship between parental mental health difficulties and CM. For instance, in the UK parental mental ill-health was identified as a significant factor in 157,600 of 496,030 (or 32%) children’s social care assessments (Department for Education. (2021), 2021). Along similar lines, in the US, a birth cohort study (n = 551,232) identified that 34.6% of infants of mothers with a mental health disorder were referred to child protective services during the first year of life (Hammond, Eastman, Leventhal, & Putnam-Hornstein, 2017).

Despite extensive research into the risk factors for CM, much uncertainty remains regarding the relationship between parental mental health (MH) and CM. This uncertainty might be, at least in part, attributed to various methodological and sociocultural factors which make comparative research on this topic challenging. For instance, there is variation across services, contexts, and studies regarding child maltreatment assessment (Stoltenborgh, Bakermans-Kranenburg, Alink, and van IJzendoorn (2015). Inconsistencies in the definition of child abuse and neglect add to the complexity. For instance, there have been changes in the use of abuse categories over time. A far larger proportion of cases were identified as physical or sexual abuse in the past compared to now, partly because in some countries (for example, Scotland (Scottish Government, 2021), new categories have been introduced (e.g. sexual exploitation or exposure to domestic violence as necessarily abusive).

There are also conceptual and methodological challenges associated with establishing parental mental ill-health. For example, studies may rely on a retrospective self-report (Sidebotham et al., 2001), a retrospective report from a child (Walsh et al., 2002, Witte et al., 2018), current symptomatology (Clement and Chamberland, 2016, Francis and David, 2008, Stearns and Cliff, 2019), current diagnosis (Davis, Matthew, Freed, & Clark, 2011), previous diagnosis (Kalebić Jakupčević and Ajduković, 2011, Kaplan et al., 2009, Whitten et al., 2020) or previous treatment of a MH condition (Ahmadabadi et al., 2018, Eriksson et al., 2016, Rodriguez et al., 2018). How mental ill-health can be classified within these categories also pose problems. The use of different reporting scales of depressive symptomatology varies between areas, with some studies electing to use the BSI (Francis & David, 2008) and others the CES-D (Clement & Chamberland, 2016; Kelley, 2015). This may result in an inconsistent attribution of diagnoses between areas.

A further issue, which is the focus of this paper, is a tendency for research to obscure the separate significance of maternal and paternal mental health for CM. For example, in a recent *meta*-analysis summarising evidence on the relationship between parenting and personality disorder, two of the eight systematic reviews, covering 211 studies, investigated mothers only while no systematic reviews investigated only fathers (Steele, Townsend, & Grenyer, 2019). Only 5% of parent respondents across the studies were fathers. Similar issues can be seen in the analysis of clinical practice, which has an overfocus of social workers on mothers (Risley-Curtiss & Heffernan, 2003)- fathers involvement is often seen as a ‘good-bad’ binary (Dominelli, Strega, Walmsley, Callahan, & Brown, 2010) with less focus on a father’s practical caring skills (Baynes & Holland, 2012). By contrast, a recent study of the relationship between employment and CM starts from the explicit assumption that fathers and mothers will play very different roles in family life and childcare, in particular, concluding that additional time with mothers (due to unemployment) was likely to reduce abuse but that the reverse was true, overall, for fathers (Lindo et al., 2018). This lack of clarity concerning the role of maternal and paternal MH for CM is despite long-recognised differences in the prevalence of mental disorders in men and women although one recent commentator wrote that ‘Sex and gender differences in mental disorders are among the most intriguing and stable findings in psychiatry. For example, differences exist regarding prevalence, symptomatology, risk factors and influencing factors, or course’ (Riecher-Rössler, 2017).

This narrative review, therefore, aims to begin to address this limitation in the research by providing an account of the empirical literature regarding fathers’ MH and CM. Specifically, it focuses on the following two research questions:

(1) What is the relationship between different dimensions of fathers’ MH and various forms of CM?
(2) How do methodological factors (e.g. self-report accounts, other informant accounts) shape the evidence about the reported relationship between fathers’ MH and CM.

The intention is neither to assume that fathers’ mental ill health makes them potential perpetrators of CM nor that mental ill health is necessarily a risk factor for CM. Rather, this work acts to summarise the evidence surrounding the association between MH and CM and utilise this evidence to suggest practical points for academics, practitioners and policymakers which will help support affected families.

## 2 Methods

### 2.1 Overview

This narrative systematic review was conducted in line with the Preferred Reporting Items for Systematic Reviews and Meta‐Analysis (PRISMA) guidelines (Moher, Liberati, Tetzlaff, Altman, & The, 2009). Searches were conducted in four databases (PubMed, PsycINFO, Web of Knowledge, Scopus) up to and including the 9th of February 2021. Search terms were sorted into four categories: (i) Population (e.g. ‘Fathers’) (ii) Fathers' MH problem (e.g. ‘mental illness’), (iii) Outcome (e.g. ‘maltreatment’), and (iv) Victim group (e.g. ‘children’). Duplicates were removed (k = 1761) before inclusion and exclusion criteria were applied to abstracts and full texts. For details of search terms and the number of results see Table 1. We conducted an update to the search on the 21st February 2023, identifying 318 papers of which 3 were relevant.

**Table 1:** Search terms.

#	Domain		Search term	PubMed	PsycINFO	Web of Knowledge	Scopus
1	Population	1	Paternal				
		2	Father				
		#	OR 1/2	47,986	51,767	132,607	110,130

2	Fathers' MH	3	Mental health				
		4	Mental disorder*				
		5	mental ill*				
		6	Depress*				
		7	Anxiet*				
		8	Psycho*				
		9	Addict*				
		10	Personalit*				
		11	Schizo*				
		#	OR 3/11	1,441,407	1,592,586	4,625,650	256,755

3	Outcome	12	Maltreat*				
		13	Exploit*				
		14	Abuse*				
		15	Neglect				
		16	“Family violence”				
		17	“IPV”				
		18	“intimate partner violence”				
		19	“Domestic violence”				
		#	OR 15/22	169,614	177,166	524,969	295,176

4	Victim	20	Child*				
		21	Adole*				
		22	Infant*				
		#	OR 20/22	1,934,657	890,806	4,847,222	2,634,715

=	Combined		Levels 1–4	602	1,550	2,628	268

MH = mental health; * denotes wildcard operator, “” indicate exact phrase/term.

### 2.2 Inclusion and exclusion criteria

**Fig. 1:**
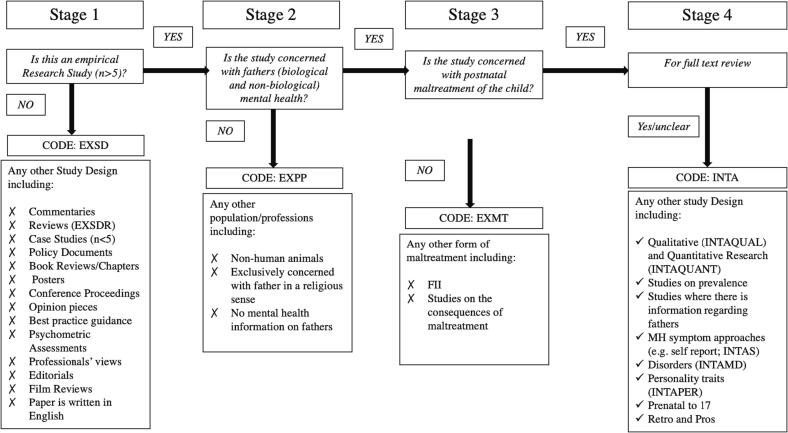
Title and abstract screening flow chart.

We included primary empirical studies on fathers’ MH and CM. We adopted a broad approach to identifying studies of CM by including studies that used both self-report retrospective accounts and studies on child abuse potential, as well as professionally substantiated assessments and referrals. Similarly, we included studies that used retrospective accounts of fathers’ mental ill-health. Both biological and non-biological fathers were included. For practical reasons, studies were brought to full-text review only if it was clear at title and abstract screening that there was specific information about fathers’ MH and CM. As such, we did not include papers that made references solely to ‘parental’ MH and CM. Due to practical restrictions, we only included studies published in English. Additionally, for this study, we did not include case studies. Here ‘case studies’ were defined as studies with fewer than n = 5 participants. A decision flow chart was used to aid title and abstract screening. This flowchart is presented in Fig. 1 and includes further details about the inclusion and exclusion criteria.

### 2.3 Full-text review

**Fig. 2:**
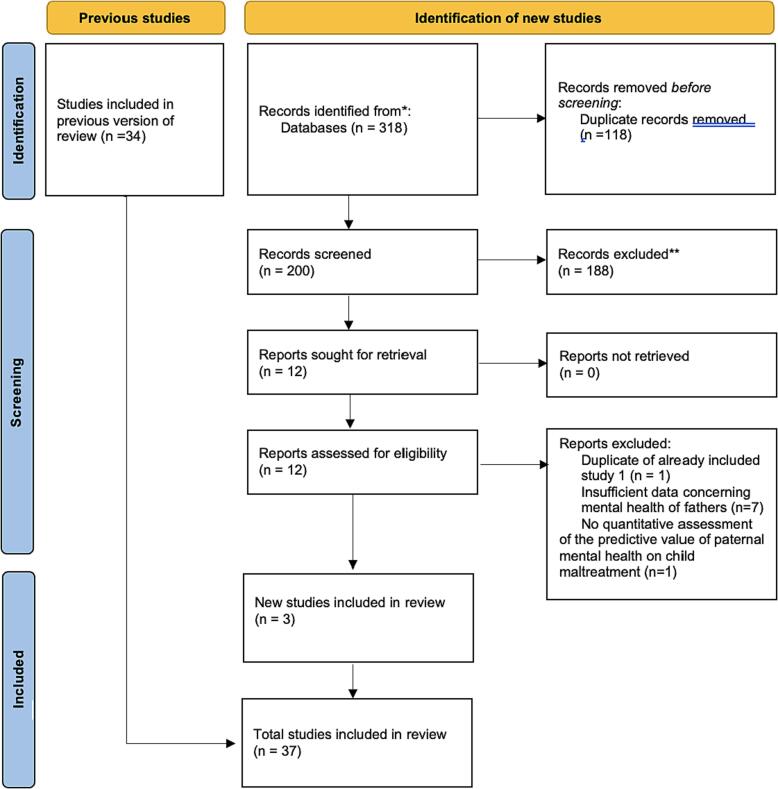
Updated PRISMA flowchart for search conducted on the 21st Februrary 2023.

In total, 151 articles were brought through to full-text review. Each paper was read in full by [Author 1] and discussed with the research team. Six papers were excluded as they were not written in English, 46 did not have sufficient data on fathers’ MH, 35 papers did not quantitatively assess the level of CM utilising fathers’ MH as a predictive value and five papers were not empirical research studies. The authors of unavailable papers were written to request the manuscript, however, 20 full-text papers identified through database searches were ultimately unable to be accessed or were not available in English. One study (Lee, Catherine, & Bellamy, 2012) was excluded because, although relevant, the reported data was duplicated in another paper which was included in the review (Lee, 2013). In total, 37 papers were included in this review (see Fig. 2 for the PRISMA flowchart). Descriptive information regarding the studies, including setting, methods and key findings, can be found in the supplementary material paper. The following was extracted from each paper by [Author 1]: Study, location, method, study design, number of children, whether the sample was limited to biological children, mental illness measured and how this was done, the outcome of interest and how this was measured, key findings, and other associated factors to CM.

### 2.4 Quality assessment

We used the Mixed Methods Appraisal Tool (MMAT) (Hong, Gonzalez-Reyes, & Pluye, 2018) to guide the quality assessment. Quality assessment was completed by two researchers. The outcome of the quality assessment is presented in Table 2. Overall, the quality of the studies was variable. Study quality is discussed through the narrative text.

**Table 2:** Quality assessment.

(See Ahmadabadi et al., 2018, Arabghol et al., 2016, Bourget, 2005, Castro-Vale et al., 2019, Čatipović et al., 2003, Clement and Chamberland, 2016, Coohey and Ying, 2006, Davis et al., 2011, Duffy et al., 2015, Eriksson et al., 2016, Francis and David, 2008, Fredman et al., 2019, Friedman et al., 2005, Garosi et al., 2023, Green et al., 2022, Haapasalo, 2000, Harris et al., 2007, Hecker et al., 2022, Kalebić Jakupčević and Ajduković, 2011, Kaplan et al., 2009, Kauppi et al., 2010, Kelley et al., 2015, Lee, 2013, Lee et al., 2011a, Lee et al., 2011b, Marleau et al., 1999, Putkonen et al., 2010, Rodriguez et al., 2018, Sachs-Ericsson et al., 2012, Schaeffer et al., 2005, Scharpf et al., 2020, Sidebotham etal., 2001, Stearns and Cliff, 2019, Takehara et al., 2017, Walsh et al., 2002, Whitten et al., 2020, Witte et al., 2018.)

Red indicates a negative response (“no”), green a positive response (“yes”), orange indicates there was inadequate information provided in the study (“can’t tell”) and grey indicates the question was not applicable.

## 3 Results

### 3.1 Characteristics of studies

A total of 37 papers fulfilled the inclusion criteria at full-text review. Seven studies used a prospective cohort design, 17 were analytic cross-sectional studies, seven used a case-control approach, five were descriptive cross-sectional studies and one utilised a mixed qualitative/ quantitative approach. Data were collected between the years 1958–2018. Note that most studies used data collected in the past two decades. However, studies that investigated filicide utilised older data to ensure sufficient case numbers due to the scarcity of the crime. Four studies utilised the Fragile Families survey, a major United States birth cohort study of babies born between 1998 and 2000 (Davis et al., 2011, Lee, 2013, Lee et al., 2011a, Lee et al., 2011b). Some studies recruited participants from similar locations, and it is possible that some fathers/ children participated in more than one study (Bourget, 2005, Clement and Chamberland, 2016, Francis and David, 2008, Harris et al., 2007, Marleau et al., 1999, Walsh et al., 2002).

Study quality was variable. Only 8 of 34 studies scored full points on the quality screen. The quality of multiple quantitative non-randomised studies was affected by the potential of non-response bias. The predominant use of retrospective study design prevented papers from concluding the presence of a temporal and causal relationship. Six papers utilised cohorts which allowed such claims to be made. Of these six studies, only one failed to find a significant relationship between the investigated forms of paternal mental-ill health and CM. Due to a descriptive, rather than analytic approach, papers investigating filicide were unable to comment on the relationship to mental ill-health and instead drew weaker conclusions on its prevalence in their sample population. Additionally, the different study design complicates comparisons of the quality of these papers compared to others. Many studies were limited in quality by classifying a lifetime diagnosis as mental ill-health being present. In addition to stigmatising those who no longer have mental ill-health, this definition limited studies as the co-occurrence of mental-ill health and maltreatment were unclear.

### 3.2 Sample population

The primary studies contained data on the parents of a total of approximately 180,000 children, approximately 7000 of whom were confirmed to have experienced maltreatment, in addition to around 21,000 who had had child protective services involvement or placement in out-of-home care. We are unable to provide exact figures because some studies applied different exclusion criteria to the same data set. Children included in the studies were aged between 0 months and 18 years. Data was gleaned from a wide range of locations. Of our studies, twenty had data from North America, two from Japan, ten from Europe, four from Oceania, one from the Middle East and two from Africa.

### 3.3 Measurement of fathers’ MH difficulties and child outcomes

Across the set of included studies, the outcome variable (i.e. ‘child maltreatment’) was operationalised in a range of ways. Seven papers used referrals to child protective services. The referral reasons included suffering from (or being at a high risk of) physical, sexual or emotional abuse and neglect. These findings are described separately, as a referral to CPS may not actually indicate maltreatment has taken place. Five studies investigated the effects of a father’s MH disorder on a father’s potential to abuse a child, as assessed with a self-report questionnaire. Seventeen papers measured the rates of substantiated CM as their outcomes. Here, substantiated implies cases where maltreatment has been confirmed by professionals and/or courts. Concerning their findings on the relationship between fathers’ MH disorders and CM, nine studies offered a descriptive analysis of a cohort of fathers where maltreatment was substantiated.

### 3.4 Fathers and negative parenting practices

#### 3.4.1 Mood disorders

19 papers explored the relationship between paternal MH disorders and CM. Of these, ten papers investigated the association with depression, two with anxiety, one with psychosis, three with post-traumatic stress disorder (PTSD) and one with either schizophrenia or bipolar. Five papers examined the effects of a range of MH disorders when assessed as an aggregate group. Of the ten papers which investigated depression, a range of types of maltreatment were investigated.

Data from six papers investigated the association between depression and physical abuse. Drawing data from the Fragile Families and Child Wellbeing study in the United States (Reichman, Teitler, Garfinkel, & McLanahan, 2001), Davis and colleagues identified that fathers with a diagnosis of depression (n = 128) were nearly four times more likely (odds ratio = 3.92, (CI = 1.23, 12.5) to report spanking their one-year-old child than fathers without depression (n = 1618; (Davis et al., 2011).In a later study, using the same dataset, Lee and colleagues (2011) found that 61% of fathers (n = 2,309) reported using corporal punishment with three-year-olds. Lee and colleagues reported that parenting stress was associated with moderate (OR = 1.22, p < 0.05) and heavy (OR = 1.53, p < 0.001) corporal punishment. Interestingly, depression was associated with heavy (OR = 1.53, p < 0.05), but not moderate (OR = 1.31, p > 0.05) corporal punishment. By contrast, generalised anxiety disorder was not associated with either moderate (OR = 0.78, p > 0.05) or heavy corporal punishment (OR = 0.71, p > 0.05).

Both studies utilised the same dataset and were of high quality yet obtained somewhat contrasting results concerning the association between fathers’ depression and child maltreatment. The difference may be explained as Davis and colleagues excluded fathers who did not reside with the child all or almost all the time while Lee and colleagues did not. Fathers who cohabited with the child had a greater incidence of moderate and heavy corporal punishment. The incidence rate of fathers who had depression varied between the two studies (7 vs 12% respectively). The weaker association between depression and physical abuse, as found by Lee et al. might be due to the inclusion of non-cohabiting fathers, who had less child contact and a higher prevalence of depression. Additionally, Lee and colleagues performed multivariate regression analysis to investigate the effect of a range of covariates on child maltreatment. In contrast, Davis and colleagues performed regression analysis only of the effect of depression whilst controlling for these covariates.

In Canada, Francis and Wolfe (2008) investigated the MH profiles of 24 fathers referred by Child Protection Services and a control group of 25 fathers recruited through the community. The results from this study indicate that fathers referred by Child Protection Services, on average, scored higher on each of the Brief Symptom Inventory (BSI) (Derogatis & Melisaratos, 1983) subscales (i.e. somatization, interpersonal sensitivity, depression, anxiety, hostility, paranoid ideation, psychoticism, and global severity index). Multivariate analysis identified an effect on mental ill-health and group membership (i.e. abusive and non-abusive; Pillai’s trace = 0.52, F(8, 36) = 3.72, p ≤ 0.01) with univariate analysis showing an association between maltreatment and somatization (*X*^2^ = 5.24, p ≤ 0.05), depression (X^2^ = 7.62, p ≤ 0.01), hostility (X^2^ = 6.34, p ≤ 0.01), paranoid ideation (X^2^ = 11.90, p ≤ 0.001), psychoticism (X^2^ = 5.63, p ≤ 0.05) and global severity (X^2^ = 6.51, p ≤ 0.01). Several sociodemographic factors including the number of children (F = 8.89, p < 0.01), marital status (X^2^ = 15.47p < 0.01), education (X^2^ = 27.35, p < 0.001, F = 15.17, p < 0.001), income (over/ under $39,000: X^2^ = 12.48, p < 0.01) and history of maltreatment as a child (F = 20.28, p < 0.01) were also associated with maltreatment, being higher in the maltreatment groups.

In the US, Kaplan et al. (2009) conducted a similar study comparing rates of depression in physically abusive (n = 99) and non-physically abusive (n = 99) fathers. Physically abusive fathers were more likely to have any lifetime depressive disorder (34.6% vs 14.3%, X^2^ = 6.97, p ≤ 0.05). The incidence of lifetime depression in mothers (χ2 = 8.10, df = 1, p < 0.01) and lifetime anxiety in fathers (χ2 = 6.59, df = 1, p < 0.01) were different in the two groups over and above parental age, education, and marital status. The quality of this study was limited by the use of any life experience of mental ill-health and insufficient control for confounders.

Two studies investigated the association between depression and neglect, with inconsistent findings. Namely, Clement and Chamberland (2016) reports a non-significant association between depressive symptomology and neglectful behaviour in children (n = 1104) at various ages: between 6 months and four years (OR = 1.5 CI-0.5–4.3), 5–9 years (OR = 3.1, CI = 0.9–9.9) and 10–15 years (OR = 2.1, CI = 0.9–5.3). Lee and colleagues found contrasting results analysing the Fragile Families survey dataset (Lee, 2013), finding that depression in fathers when the child was three years old more than doubling the odds of neglect when the child was five (n = 1089, OR = 2.04 (1.22–3.42)). Both studies were of high quality and controlled for socioeconomic status, the former with the use of perceived economic situation and the latter comprehensively with a range of measures of economic hardship. One factor that may have contributed to inconsistent findings was that they operationalised neglect differently: a wider range of questions were asked to parents to assess neglect by Clement and colleagues than by Lee.

Three papers gave details on the demographic profiles of children and fathers who are more likely to be affected as a result of a father’s depression (Lee, 2011; Stearns and Cliff, 2019, Takehara et al., 2017). In one paper, investigating retrospective reports of fathers’ depressive symptoms and abuse from students at a university in the United States, reports of father’s depressive symptoms predicted reports of psychological maltreatment in daughters (n = 662 ß=.22p < 0.05) but not sons (n = 350), and physical maltreatment in both sons (n = 350, ß=0.19, p < 0.05) and daughters (n = 662, ß=0.22, p < 0.05) (Stearns Cliff, 2019). In a study carried out in Japan, a non-significant association in CM practices with 2-month-old babies was found in fathers who had prenatal (n = 183, OR: 1.62 [95% CI: 0.41–6.44) or prior postnatal (n = 183, OR = 3.67 [95% CI: 0.97–13.66) depression, while greater odds were observed in fathers with current depression (n = 181, OR: 7.77 [95% CI: 1.83–33.02]) (Takehara, 2017). This indicates that the more recent the depression, the higher the chance of maltreatment practices. It is worth noting, however, that the predictive scale used to measure ‘CM tendency’ in this study has not been validated for fathers and included criteria which would not necessarily indicate maltreatment. For instance, the scale gives the same weighted score to parents who hit their child as those who leave their child alone in a car. Additionally, the quality of this study was limited by incomplete outcome data.

The third study utilised data from the Fragile Families study to investigate the associations between paternal major depression when a child was three years old, the birth country of the father and the father’s aggression when the child was 5 years old. The father having a major depression diagnosis when a child was 3 did not predict physical (n = 372, ß = 0.02, p > 0.05) or psychological (n = 372, ß = 0.23, p > 0.05) aggression when the child was 5. A father's country of birth predicted the use of physical (ß = 0.25, p < 0.01) and psychological (ß = 0.24, p < 0.05) aggression when the child was five years old (Lee, 2011).

Several papers investigated the association between a father’s anxiety and substantiated maltreatment. In a recent study, Stearns Cliff (2019) reported that fathers' anxiety symptoms predicted psychological maltreatment of sons (n = 350, ß=0.19, p < 0.05) but not daughters (n = 662) and did not predict physical maltreatment (Stearns Cliff, 2019).

Two studies conducted in the United States investigated the association between fathers’ anxiety and physical abuse, with conflicting results. The first identified higher levels of anxiety symptoms amongst fathers who had been referred to child protective services due to a report of physical abuse (19.2%, n = 52) compared to those who had not been (2.9%, n = 70) (Kaplan et al., 2009). In addition, any lifetime anxiety diagnosis of a father was significant over and above parental age, education, and marital status (χ2 = 6.59, df = 1, p < 0.01) between the 2 groups. The use of any lifetime diagnosis as a measure however limits findings, as the co-occurrence of mental ill-health and maltreatment cannot be confirmed decreasing the confidence of a causal relationship. In the second study, a diagnosis of anxiety showed no significant effect on moderate (n = 2260, OR = 0.78, CI = 0.39–1.55) or heavy (n = 2260, OR = 0.71, CI = 0.32–1.58) corporal punishment (Lee, 2011). These findings differ perhaps in part due to differences in measurement. Whereas Lee and colleagues used the CIDI-SF (Kessler, Andrews, Mroczek, Ustun, & Wittchen, 1998), Kaplan et al used the presence of a current or lifetime psychiatric disorder of fathers as assessed with the structured clinical interview for DSM-III-R (Spitzer, Williams, Gibbon, & First, 1992). Further, Kaplan compared rates of anxiety in parents of children who had and had not been reported to child protective services due to physical abuse whilst Lee et al measured the corporal punishment frequency as per a self-report form. Due to these factors, direct comparisons cannot be made.

Together these studies provide tentative evidence for an association between paternal mood disorders and child maltreatment. Yet it is important to note that there is variation between these studies for the assessment of both mental health and negative parenting practices. Another source of variation is that studies took an inconsistent approach to controlling for covariates and examining moderators. Therefore, although the balance of evidence would seem to suggest that paternal mental health is associated with an increased risk of CM, much remains unclear about the nature and strength of the association.

#### 3.4.2 Aggregate measures of mental health and child maltreatment

Five papers investigated the combined effect of a range of different MH disorders (i.e. by creating an aggregate dichotomous mental ill-health variable) on the rates of CM, again with mixed results. First, using administrative CPS, hospital and social care records, Haapasalo (2000) investigated the rates of paternal mental ill-health in a group of Finish males (n = 78) living in a young offenders institution. Data was obtained from the CPS, social services, clinic, and hospital records of these individuals-covariates included spousal, physical and psychological abuse, maternal MH, a paternal and maternal conviction for a crime, maternal and paternal alcoholism, financial problems and neglect. This study did not identify a significant relationship between a father’s mental ill-health and neglect, physical, or psychological abuse (Haapasalo, 2000). These findings are at variance with a later small study in the United States, which reported that families with a father with a MH disorder had a large increased likelihood of neglecting their child for over (n = 43), compared to under two years (n = 46) (ß = 2.96, SE = 1.41, odds ratio expected ß = 19.38) (Coohey Ying, 2006). While both studies were of high quality, the limited literature and small sample sizes prevent a sufficient comparison from being made.

Two studies investigated the relationship between self-reports of adults on their experiences of CM, and reports of MH disorders in their father (Walsh et al., 2002, Witte et al., 2018). Both studies showed that having a father with a reported MH disorder resulted in increased reports of physical abuse made by the adult child: Witte et al reported an OR = 3.96 and Walsh et al reported OR = 2.4. Further, Witte et al. (2018) reported that fathers' MH disorders increased the incidence of sexual abuse (OR = 2.3 (CI = 1.4–3.6) as well as emotional abuse (OR = 4.38 [1.93, 9.93] p < 0.001), physical neglect (OR = 4.92 [2.24, 10.78] p < 0.001) and emotional neglect (OR = 3.79 [1.75, 8.20], p < 0.01). In both studies, it was unclear whether there was a co-occurrence of paternal mental ill-health and maltreatment. The strength of the conclusion of Witte et al. is further limited by an incomplete, non-representative sample and confounders being insufficiently accounted for.

Using a different approach, Ahmadabadi and colleagues reported findings from a sample of 2064 participants from an Australian birth cohort study (Ahmadabadi et al., 2018). A questionnaire asked if a father had had treatment for a MH disorder by the time a child was 14. This was used in conjunction with the adult child completing the Child Trauma Questionnaire at the age of 30 to retrospectively assess for the presence or absence of abuse. While of generally high quality, the quality assessment did identify a lack of complete outcome data. One of the key findings from this research was the mother’s partner having been treated for a MH problem predicted sexual abuse (OR = 2.20 (CI = 1.48, 3.25)) and neglect (OR = 1.92 (CI = 1.33, 2.77)) in daughters and emotional abuse (OR = 2.06 (CI = 1.03, 4.12)), physical abuse (OR = 2.21 (CI = 1.22, 3.98)) and neglect (OR = 2.35 (CI = 1.51, 3.68)) in sons. A multivariate model indicated that, in sons, the mother’s partner having been treated for a MH problem confounded the association between maternal intimate partner violence victimisation and physical abuse.

Across these five studies, the varied effects of different MH disorders, combined into one category, may contribute to the range of results. Further variation will likely occur due to multiple studies lacking distinction between current and previous MH conditions of the father. Indeed, one study found that it was only current, not past, MH difficulties that predicted maltreatment of young babies (Takehara, 2017). Therefore, although there may be some indications that broadly defined mental health difficulties are associated with child maltreatment, the strength of this association remains unclear.

#### 3.4.3 PTSD, psychosis and child maltreatment

Three studies explored trauma-related disorders such as PTSD and their impact on CM. The first of these utilised structured interviews which took place in a refugee camp in Western Tanzania. Symptoms of PTSD were assessed by the brief symptoms inventory and maltreatment was assessed utilising the child-parent conflict tactics scale (Scharpf, 2020). In this study, PTSD symptoms were not significantly correlated with maltreatment (n = 226, intercorrelation = 0.08, SE = 0.09, p > 0.05). In a follow-up study with this sample, Hecker and colleagues (2022) report that although paternal PTSD and depression were not significantly associated with increased rates of child-reported child violence (p > 0.5), the authors did observe a significant association between paternal PTSD and maternal self-reported child violence (β = 0.16). Another study used the childhood trauma questionnaire (short-form) to compare the childhood experiences of Portuguese children whose veteran parents did (n = 46) and did not (n = 15) have PTSD (Bernstein et al., 2003). A lifetime diagnosis of PTSD had no significant association with emotional abuse, sexual abuse, physical abuse, emotional neglect, or physical neglect (Castro-Vale, 2019). Insufficiency of both complete outcome data and control for confounders limited the strength of this latter finding.

Only two studies investigated fathers with psychosis and schizophrenia. The first of these was an earlier study (Francis & David, 2008), comparing 24 abusive fathers were compared to 28 controls. This study reported that more abusive fathers had psychotic symptoms when assessed utilising the brief symptoms interview (42.9% vs 10%, X^2^ = 5.63, p < 0.05). The second was a more recent study conducted by Garosi and colleagues (2023), exploring the association between paternal mental health issues and schizophrenia outcomes. A multivariate analysis reported that children whose fathers had schizophrenia or bipolar disorder reported increased physical abuse (aOR = 0.081, p = 0.042) and emotional abuse mean score (aOR = 0.146, p < 0.001).

The evidence regarding the association between paternal trauma disorders, psychosis and CM is limited and thus large gaps remain in our understanding of the associations between these phenomena.

### 3.5 Papers investigating child abuse potential

Five papers investigated the association between fathers’ MH disorders and the potential to commit child abuse. In general, these papers reported a stronger association than the papers which measured substantiated maltreatment (see above). Fathers' depression symptoms were shown to be associated with an increased child physical abuse potential in three independent papers. Two of these papers reported cross-sectional associations (ß = 0.38, p < 0.001, n = 85 (Kelley, 2015), ß = 0.55, p < 0.01, n = 175 (Schaeffer, 2005)). Another paper reported from a longitudinal study (ß = 0.239, p < 0.038, n = 151) (Rodriguez, 2018). In other papers, fathers’ PTSD symptom severity and diagnosis were explored, both showing associations with a higher potential to commit child abuse. PTSD symptom severity was correlated with increased physical abuse potential (Fredman et al., 2019) (intercorrelation = 0.53, P < 0.001, n = 150). Concurrently, fathers with a diagnosis of PTSD (n = 30) had a higher potential for physical child abuse than parents from the general population (n = 100) ((333.2 + −18.0) vs (79.61 + −9.9)) (Kalebić Jakupčević and Ajduković, 2011). The Child Abuse Potential Inventory, which measures physical child abuse potential, was utilised as the sole measure in four of these five studies. Four of the studies measured the risk of physical abuse alone as an outcome.

Insufficient control for covariates limited the quality of two of these papers (Kalebić Jakupčević and Ajduković, 2011; Kelley, 2015). Otherwise, the papers were of high quality. However, it is important to note that child abuse potential is a distinct phenomenon from substantiated child abuse. Therefore, although these studies suggest a statistically significant association between certain mental health conditions and potential child abuse, the available evidence is not sufficient to make population-level claims about the magnitude of this association, and a sharp distinction should be drawn between potential and substantiated abuse.

### 3.6 Papers investigating CPS involvement

Conflicting evidence was found in six cohort studies that assessed risk factors for CPS involvement. Using a large data set collected in Avon, Sidebotham et al., 2001 found that fathers’ symptoms of depression tripled the odds of contact with child protection services (OR = 3.6, 95% CI 1.63–7.96, P < 0.005) A paternal history of depression was found in 9 of 162 registered children (24.3%), compared to 495 of the 13,976 non-registered children (6%). In a study previously described, a diagnosis of depression when the child was 3 years old doubled (OR = 2.42, CI = 1.05–5.58, p < 0.01) the chances of contact with child protection services by the time the child was 5, though the confidence interval was wide (Lee, 2013). These findings are at variance with some of the work of Kaplan et al. (2009) who reported that diagnosis of depression in fathers did not predict that a family would have been referred to CPS services (n = 52 abusive fathers, 70 non-abusive controls). This may suggest that a diagnosis of depression has either differing effects on different types of abuse, or that it has differing associations depending upon whether a CPS report is substantiated. Both paper’s quality was limited by the completeness of outcome data. At a population level, the evidence is not sufficient to support strong claims.

Papers analysing fathers who were identified as having any form of MH disorder also gave mixed results. One paper investigated the association between the presence of a diagnosed paternal MH disorder and contact with CPS before the child was 14 (Whitten, 2020). The study utilised a large sample size from administrative records from New South Wales (n = 71,661) with 16,585 children who had had contact with child protective services. Overall, a diagnosis of an ICD-10 AM MH disorder had little impact on early contact with child protective services (hazard ratio = 1.05 (1.00, 1.10)). Another study investigated reports from families in the ‘nurturing family’s network’, Connecticut's child abuse and neglect program (Duffy, Marcia, Andrea, & Leventhal, 2015). Families had either a substantiated or unsubstantiated report made to the CPS. The relationship between a paternal history of mental ill health, as assessed from CPS reports, and whether the claim would be substantiated was investigated. It was found that paternal MH history caused a non-significant increase in the chances that a claim would be substantiated (n = 65, OR = 2.22 (CI = 0.56, 8.77 )). These findings however are at variance with the results of a large birth cohort (n = 72,059) study conducted by Green and colleagues (2022) in Australia which reported a positive association between paternal mental health at birth and child maltreatment by the age of 14 (OR = 2.17 (CI = 1.77–2.65).

One explanation for the variation between studies might be that more impairing mental health difficulties might have a stronger association with child maltreatment than common mental health difficulties. If this is the case, then these effects could be weakened when a broad category of mental health is used. From a study design perspective, the use of CPS involvement as a measure of child abuse has several appealing qualities. For example, due to the need to maintain records throughout, many services will have comprehensive information on many individuals, allowing a large amount of outcome data to be accessible to the investigator, with linked information as to when this outcome occurred. Additionally, it may be that a father’s MH is mentioned earlier compared to when a form of abuse is reported, indicating that a MH disorder was likely present before the referral occurred. This allows stronger conclusions on the causal relationship between a father’s MH and CM to be made. However, it is also important to consider the data in light of some of the following limitations. Families on the CPS registers were less likely to respond to surveys compared to other members of the cohort (Sidebotham et al., 2001). Additionally, the use of CPS reports introduces bias, as they may overestimate or underestimate rates of child abuse or fathers’ MH disorder. For example, the greater contact of families in prevention programs with reporters compared to non-participating families may result in overestimation because of surveillance bias (Chaffin, 2006). One source of underestimation may come from the fact that a lack of data within CPS reports was more often seen for fathers than for mothers (Duffy et al., 2015), with a male partner’s response rate ranging between 14 and 34% in the findings of Sidebotham and colleagues. Another source of underestimation may be inferred from the finding that only 7.6% of individuals in the community who report being maltreated as a child reported coming into contact with child protection authorities (Afifi et al., 2015).

The suggestion of underdiagnosis is additionally seen in two studies assessing fathers’ psychopathology in a group of children who were confirmed sufferers of more commonly occurring types of maltreatment. Investigating records of reported child abuse from Bjelovar Croatia, the first of these studies investigated whether MH treatment of the father had been documented in police reports as an index of the psychopathology in fathers. Relatively low levels were reported, for example, in the 24 cases of fathers’ emotional abuse, not one father had received treatment (Čatipović, Kudumija-Slijepčević, & Novalić, 2003). The second of these studies conducted diagnostic interviews with fathers (n = 66) following a clinician’s diagnosis of child abuse and neglect in a hospital in Tehran. Here, when diagnostic interviews were conducted with fathers whose children had been abused, there were higher levels of reported psychopathology: 4 (6%) were diagnosed with general anxiety disorder, 4 (6%) with depression, three (5%) with ADHD and two (3%) with OCD (Arabghol, 2016).

### 3.7 Filicide

Seven studies provided descriptive accounts of psychopathology among fathers who had killed at least one of their children. These studies reported generally high levels of MH disorders. It is of note that cases of filicide represent such a small set of examples of CM that these papers utilise data from as far back as 1958 to get sufficient numbers of filicides (Friedman, Houda, Holden, Noffsinger, & Resnick, 2005). First, a Canadian study investigated the Coroner’s reports of all filicides occurring in Quebec between 1991 and 2001 (n = 60). Severe psychopathology, as reported by such a MH condition being reported in either the coroner's case files or linked medical records, was determined in 36 (60%) of the fathers. 31 (52%) fathers had major depression and 6 (10%) had schizophrenia (Bourget, 2005). A study in the United States also investigated Coroner’s reports, analysing the Cuyahoga County coroner’s records of filicide-suicide (i.e. when a father kills the family and dies by suicide) which occurred between 1958 and 2012 (n = 20). The presence of a MH condition was again defined as per the coroner’s report. It was found that 15 (75%) of these men had a MH disorder. Five (25%) had psychosis, 10 (50%) had depression and four (20%) had delusional disorder (Friedman et al., 2005).

Three studies assessed MH of a father by a clinical interview conducted after the act of filicide, finding an increased prevalence of MH disorders. Analysing results of clinical interviews from all filicide offenders in Austria and Finland between 1995 and 2005 (n = 45), it was found that 95% of fathers had a diagnosis according to DSM-III criteria. Three per cent of fathers were diagnosed with psychotic disorder, 30% with non-psychotic depression and 57% with personality disorder (Putkonen et al., 2010). A similar study analysed Finnish fathers, who committed filicide between 1970 and 1994 (n = 15). All 15 fathers received a diagnosis of a psychiatric disorder. Of the 15 that underwent assessment, two had psychosis, one had psychosis and substance abuse disorder, one had depression and substance abuse disorder/ personality disorder, four had personality disorder, five had personality disorder and substance abuse disorder and two had personality disorder features (Kauppi, Kirsti, Karl, & Tuija, 2010). The final of these three studies utilised data from psychiatric, psychological and criminological assessments found in the files of filicidal men in a Canadian inpatient facility between 1982 and 1994. All 10 were diagnosed with either an axis I or axis II disorder: four had mood disorders, 8 had personality disorders, 4 were psychotic at the time of the offence, and 1 had schizophrenia (Marleau et al., 1999).

The final study utilised data from the Australian homicide project which interviewed perpetrators of murder and manslaughter offences. They reported that none of the filicidal fathers (n = 9) described had a MH diagnosis (Eriksson et al., 2016). Two factors influence the strength of this finding. Firstly, the small sample size. Secondly, this study assessed fathers’ MH by asking whether the father had received MH treatment or assessment in the year up to the murder, which likely represents a considerable underestimate of MH needs. In Austria and Finland, while 95% of fathers received a diagnosis on the DSM-III classification post-filicide, not one of them had received an official diagnosis pre-filicide (Putkonen et al., 2010).

Fortunately, filicide is rare and thus studies on this phenomenon usually involve retrospective accounts of paternal mental health. Together, this literature does not provide strong evidence that mental health is a particularly potent predictor of filicide.

## 4 Discussion

In research and practice, parental mental health has become embedded as a key risk factor for CM. Yet much remains unclear about this association, particularly concerning fathers. This narrative review documents the available evidence on the association between paternal mental health and CM. Synthesising the literature, the association of different mental illnesses were discussed concerning (a) substantiated maltreatment; (b) potential to commit maltreatment; (c) CPS referrals and (d) filicide. Although this review included 34 studies, there was considerable variation between studies regarding the conceptualisation of mental health and the assessment of child maltreatment. Overall, this review identifies large gaps in our knowledge regarding the association between different MH difficulties and CM in fathers.

### 4.1 Comparison with the literature

The evidence from several, but not all studies suggests that there may be a significant association between paternal depression and physical punishment or physical abuse. Although this finding is consistent with the literature on maternal depression and CM (Conron, Beardslee, Koenen, Buka, & Gortmaker, 2009) and the broader literature on maternal CM (Dubowitz et al., 2011, Kim et al., 2014), a note of caution is due regarding the magnitude of these associations. Several of the studies included in the current review reported statistically significant results without effect sizes and others (e.g. Takehara, 2017) reported substantial effect sizes (OR = 7.77), but with wide confidence intervals (95% CI: 1.83–33.02). Therefore, even though the balance of evidence suggests that depression is associated with CM, beyond that, much remains unclear about the strength of this association. Throughout, caution must be exercised when translating the findings of studies in one country to different cultural, societal and child protection contexts.

Even less is clear about whether there is an association between other MH difficulties such as anxiety and PTSD. For instance, in three studies, PTSD symptoms and diagnosis were found not to be significantly correlated nor predictive of substantiated CM. However, PTSD symptoms were associated with fathers’ potential for physical abuse as measured by the Child Abuse Potential Inventory (CAPI). A lack of control of confounders limited the quality of both studies investigating substantiated CM. While fathers’ anxiety was shown to be non-significant to CAPI scores, lifetime fathers’ anxiety disorder predicted physical abuse. Furthermore, fathers with PTSD symptoms had a much higher potential for child abuse compared to parents with mixed anxiety and depressive disorder (Kalebić Jakupčević and Ajduković, 2011), which is in contrast to most of the studies reviewed. It is important to consider potential reasons for the differences in results seen with substantiated abuse and with CAPI scores. This might be partly explained by the psychometric properties of the CAPI. Although the CAPI has been shown to have good internal consistency in general populations (Ellonen, Rantanen, Lepistö, Helminen, & Paavilainen, 2019), a German study found that confirmatory factor analysis to assess factor structure was confirmed for mothers but failed for fathers who completed the brief form of the CAPI (Liel, Steinert, Kindler, Lang, & Eickhorst, 2019).

Another explanation might be how PTSD was measured. This speaks to another key conceptual challenge in research on this topic. Namely; the use of a lifetime diagnosis (as opposed to a 12-month incidence) may not sufficiently separate at risk from other factors. A core challenge with these measures is that they do not delineate between treated and untreated MH conditions. The coupling of lifetime and 12-month incidence therefore might substantially influence the magnitude of effect sizes and ultimately hamper efforts to identify mechanisms. Going forward we suggest that future research on this topic where possible (a) reports effect sizes and confidence intervals, (b) delineates between different measures of maltreatment and (c) conducts subgroup analysis on lifetime versus 12-month incidence measures.

**Fig. 3:**
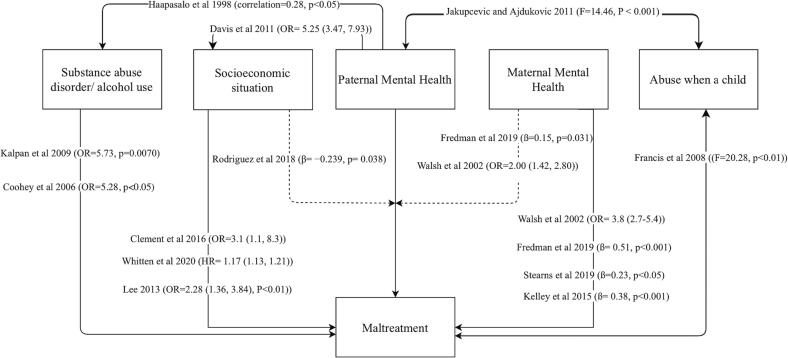
Diagram showing a selection of covariates and their interactions as found by papers included in the review.

Additionally, to develop a full picture of the association between paternal MH and CM, additional studies will be needed to provide an account of relevant covariates and moderators. Fig. 3 shows the covariates which reviewed papers found to contribute both to maltreatment and also to either fathers’ mental ill health or the relationship between fathers’ mental ill health and maltreatment. Effect sizes have been converted to a common coefficient whenever possible. Maltreatment is used as a broad term in this diagram. Some papers from the review identify that different factors may contribute differently to different forms of maltreatment, but overall, the literature reviewed is not mature enough to permit such specificity. In addition, for simplicity, this diagram considers all MH conditions under the same category, which is not reflective of the interactions of these covariates in a real-life scenario. Where more than one number was available in a paper, the largest is used in Fig. 3.

Studies reported that socioeconomic factors were associated with child abuse (Lee, 2013), contact with CPS (Lee, 2013, Whitten et al., 2020), and child abuse potential (Kalebić Jakupčević and Ajduković, 2011). Many examples of this association can be found in the literature (Coulton et al., 1995, Drake, 1996, van IJzendoorn et al., 2020). The association between fathers’ psychotherapy and parent–child aggression was shown to be stronger in parents who had two of the following factors: public financial assistance, incomes 150% below the poverty line, a high school education or less, aged 18 or younger (Rodriguez, 2018). Unemployment was nearly four times higher in fathers with depression (Davis et al., 2011). Socioeconomic factors have been found elsewhere to have direct and indirect effects on maltreatment. Income inequality has a direct, non-linear effect on predicting state care rates. Economic status is a risk factor for fathers’ postpartum depression (Philpott, 2018), and it has been shown to mediate the effect between fathers’ depression and hostility (Reeb, J., & Martin, 2013).

Poverty may be more associated with forms of maltreatment such as neglect (Isumi, Takeo, Nobutoshi, Manami, & Tsuguhiko, 2018) for example, by preventing a parent from meeting a child’s basic needs due to lack of money (Berger, 2011). Direct interventions for poverty and neglect, such as financial support or the use of social welfare resources have been argued to be required to combat neglect (Isumi et al., 2018, van IJzendoorn et al., 2020) and other forms of maltreatment (Spencer et al., 2021).

Several studies found positive associations between the fathers’ MH and substance or alcohol abuse (Conner and Gamble, 2009, Grant et al., 2004, Haapasalo, 2000). This association may both directly and indirectly mediate the association between fathers’ psychopathology and CM. Extensive research shows the direct association between fathers’ alcoholism and CM/ child abuse potential (Ammerman et al., 1999, Chaffin, 2006, Lee et al., 2011b). Mood swings, irritability and mood disorders stemming from substances and alcohol use (Kessler, 2004), may suggest a pathway by which substance use disorder can lead to increased psychopathology symptoms, and in turn CM.

Parents with mental mixed anxiety and depressive disorder or PTSD reported higher levels of abuse from their parents (Kalebić Jakupčević and Ajduković, 2011). This reflects reports from the literature that those who suffer from child abuse are more likely to later suffer from mental health conditions (Adams and Knight, 2018, Scheuer et al., 2018). A wide range of literature both within (Francis and David, 2008, Kalebić Jakupčević and Ajduković, 2011) and outside the scope of this review (Font et al., 2020, Mulder et al., 2018) show that maltreating parents were more likely to be maltreated as a child. Taken together, along with the overarching findings of this review, this may suggest that subsequent depression of the child may represent one way that intergenerational transmission of CM is mediated in males. Literature concerning maternal MH and CM supports this - maternal depression has been shown to mediate the association between a history of childhood abuse in mothers and subsequent CM (Choi et al., 2019, Kim et al., 2014).

### 4.2 Limitations of findings and implications for future research and clinical practice

Further research is needed on this topic to have sufficient knowledge to effectively translate to clinical practice. Insufficient data is available for a range of MH disorders, which prevents sufficient conclusions from being drawn. Only six papers investigated anxiety, four papers investigated PTSD, and only one examined psychotic symptoms. Depression is a common MH problem and thus it makes sense it is the focus of much of the literature (Subramaniam et al., 2019, Zeng et al., 2019), still much more investigation is needed concerning less common MH disorders. Assuming equivalent relationships between different MH disorders and their resultant effects on maltreatment, aggregating them as “any report of a MH disorder”, results in an insufficiently detailed analysis of less prevalent disorders. In the future, the use of larger cohorts or administrative data from MH or social services may offer greater statistical power and overcome the need for such aggregation. There is insufficient information in the analysed papers to draw effective conclusions on the potentially different effects of biological and non-biological fathers’ MH on CM. As patterns of maltreatment behaviour differ between these two groups (Berger and Waldfogel, 2009, Harris et al., 2007, Radhakrishna et al., 2001), the contributing risk factors likely do also. This may form a topic of future research.

For non-depression MH disorders, the lack of certainty of evidence described concerning their association with maltreatment comes in sharp contrast to the findings on the effects of a range of confounding factors. While depression had a more consistent association, the relative effect sizes of these were rarely the largest reported. It may be that fathers’ mental ill health directly increases the likelihood of maltreatment. Alternatively, mental ill health may exacerbate previously existing risk factors involved in CM, resulting in a positive feedback cycle due to a network-like association with other contributing factors (Fig. 3). While this network results in large amplifying effects due to small changes in fathers’ mental ill health, it allows policymakers a wide range of potential interventions to break the chain.

Imbalances in the literature need to be addressed. The comparatively greater evidence base concerning maternal MH, and its association with CM, prevents sufficient analysis of the whole situation. The lack of focus on fathers means that recommendations for both policymakers and clinical practitioners may miss essential detail required to translate academic findings into positive, ground-level effects. In addition to the imbalance concerning parents, an imbalance in the type of abuse investigated also needs to be addressed. In England, neglect is the most common type of abuse documented in child protection planning (48%; Department for Education, 2021). In the USA, the proportion of CM cases attributed to neglect is even higher (Bywaters and Skinner, 2022). Only eight studies considered the relationship of fathers’ MH to neglect as an outcome. This compares to 21 studies which utilised physical abuse or the potential thereof as an outcome. Six studies investigated sexual abuse, and six reported fathers’ characteristics in the cases of filicide – despite the extreme rarity of such acts. These proportions are not comparable to a nationally representative sample of different types of child abuse rates. The large number of papers choosing metrics such as physical abuse or filicide may represent a bias in the literature: fathers (especially those already stigmatized due to mental illness and associating factors) are more likely to be presumed to be physically abusive, compared both to other forms of abuse, and those fathers who are not abusive at all. Questions could also be asked assessing the difference between biological and non-biological fathers. Still, it is important to note that different forms of abuse may co-occur and thus it may be problematic to consider one form of abuse without examining co-occurrence.

However, the nature of the questions asked could also diversify to better reflect the real-life situation experienced by those with whom this research is concerned. While a negative effect of fathers’ mental ill health was found in some studies, this finding is impacted, and potentially biased by research being based on the presence of a father’s mental ill health as a risk factor, rather than the treatment or support of such as a protective factor for CM. The goal of the research field is to protect children from maltreatment. If mental ill health affected a tiny number of fathers and had traumatic effects on their children then this approach may be warranted. However mental ill health is experienced by many fathers throughout their children’s lives. Research should aim to use outcomes which more appropriately reflect this. These research questions risk stigmatising fathers experiencing mental ill health, rather than shaping decisions of how best to support them in their task of parenting their children. Much more data is needed concerning the outcomes of supporting fathers. While the questions which still exist as to individual MH condition’s effect on maltreatment indicate a need for further research into this, it is important not to neglect many of the findings which have been highlighted concerning the effects of various confounding factors. Such an approach will need to sensitively consider the lack of trust of those with a position of societal authority many affected men may have, which will likely include academics.

Finally, from a methodological point, two limitations are relevant. First, we focused on papers that included paternal mental health and not those that included direct comparisons between parents or indeed other family members. We regard this as a crucial area for further work. Second, although the review was systematic and the research questions remained consistent throughout, it was not pre-registered. The review protocol can be obtained by contact with the corresponding author.

## 5 Conclusion

In research and practice, parental mental health has become embedded as a key risk factor for CM. Yet much remains unclear about this association, particularly for fathers, reflecting both insufficient good quality, detailed and diverse research and inadequate theorising about the nature of the MH and CM relationship. This review documents the available evidence on the association between paternal mental health and CM. The evidence from several but not all studies suggested that there is a significant association between paternal depression and physical punishment or physical abuse. Beyond this, however much uncertainty exists, including the magnitude of any association. Limited and conflicting evidence was also identified in relation to depression and other forms of CM. Similarly, we found limited and conflicting evidence regarding anxiety and all forms of CM. Other types of mental health difficulties received scant attention with a few studies exploring other conditions such as PTSD, psychosis, and personality disorders. Paternal mental health was commonly identified in the literature on filicide. However, given the extremely rare nature of filicide, coupled with the fact that this literature is dominated by retrospective descriptive studies, the inferences that can be drawn from this literature are limited. Various covariates and moderators warrant close attention in future studies. Although this review included 34 studies, there was considerable variation between studies regarding the conceptualisation of mental health and the assessment of child maltreatment. Taken together, however, the findings from this review suggest that the available evidence base is not sufficient to draw firm conclusions about the association between different MH difficulties and CM in fathers.

## CRediT authorship contribution statement

**Ian Holdroyd:** Conceptualization, Methodology, Writing – original draft. **Paul Bywaters:** Conceptualization, Methodology, Writing – review & editing. **Robbie Duschinsky:** Conceptualization, Methodology, Writing – review & editing. **Taurean Drayak:** Writing – review & editing. **John Taylor:** Writing – review & editing. **Barry Coughlan:** Conceptualization, Methodology, Writing – review & editing.

## Declaration of Competing Interest

The authors declare that they have no known competing financial interests or personal relationships that could have appeared to influence the work reported in this paper.

## Data Availability

The authors are unable or have chosen not to specify which data has been used.
