# Supplementary material for: Fathers' mental Ill-health and child maltreatment: A systematic review of the literature
Source: Child Youth Serv Rev. 2024 Feb;157:107317. doi: 10.1016/j.childyouth.2023.107317 (PMC10847972; doi:10.1016/j.childyouth.2023.107317)
Supplement: Supplementary Data 1 [file mmc1.docx]

S1. Description of included studies

| **Study** | **Location and method** | **Study design** | **n** | **Biological or non-biological fathers?** | **Mental health condition (method of assessment)** | **Outcomes (method of assessment)** | **Key findings** | | **Any other associations?** | **Age** | **Control of cofounder** |
| --- | --- | --- | --- | --- | --- | --- | --- | --- | --- | --- | --- |
| Ahmadabadi (2018) | Utilised the Queensland study of pregnancy. Mothers completed a questionnaire at their first antenatal visit and at follow ups when their child was 5 months and 14 years old. Their child completed a questionnaire when they were 30 years old. | Cohort | 846 fathers  846 children with sufficient data on fathers  178 maltreated children | Both | Whether at the 14 year follow up, the Mother’s partner had been treated for a mental health problem. | Sexual Abuse  Physical Abuse  Emotional Neglect  Physical Neglect  (Assessed by the childhood trauma questionnaire completed by the 30-year-old child) | In univariate analysis, the mother’s partner having been treated for a mental health problem significantly predicted sexual abuse (OR=2.20 (CI=1.48,3.25)) and neglect (OR=1.92 (CI=1.33, 2.77)) in daughters and emotional abuse (OR=2.06 (CI=1.03, 4.12)), physical abuse (OR=2.21 (CI=1.22, 3.98)) and neglect (OR=2.35 (CI=1.51, 3.68)) in sons.  A multivariate regression model was built to model the covariates which mediate the relationship between maternal intimate partner violence victimisation and child maltreatment. In sons, the mother’s partner having been treated for a mental health problem confounded the association between maternal intimate partner violence victimisation and physical abuse. | Mother’s partner being unemployed significantly increased predicted emotional abuse, (OR=1.61 (CI=1.16, 2.24)) sexual abuse (1.48, (CI=1.06, 2.05)) and neglect (OR=1.52 (CI=1.13, 2.04)) in daughters but did not result in a significantly increased rate of any form of maltreatment in sons. | | 0-18 | Covariates in multivariate analysis included race, residency problems, family income, mother’s education, mother’s and mother’s partner’s employment, family social network, mother’s age, mother’s marital changes, if partner was the biological father of the child, length of current relationship, number of children, maternal depression and child behavioral problems. |
| Arabghol (2016) | Children in a hospital in Tehran who were diagnosed by a clinician as suffering from child abuse and neglect. | Cross sectional | 73 children. 66 parents (66 mothers and 66 fathers) were investigated. There were 56 cases of physical abuse, 53 cases of emotional abuse and 3 cases of neglect. | Information not found. | Diagnosis of mental health disorder based on DSM-IV and SADS following diagnostic interviews of the parents. | There was no measurable outcome with respect to maltreatment. | 13 fathers were diagnosed with substance use disorder, 4 with general anxiety disorder, 4 with depression, 3 with ADHD and 2 with OCD. |  | | Mean 6.90, | N/a |
| Bourget (2005) | Investigated cases of paternal filicide in Quebec occurring between 1991-2001. Examined the coroner's files from these cases. | Cross sectional | 60 paternal perpetrators and 77 victims. | Information not obtained | Any mental health condition reported in the coroner's case files, or medical records when appropriate. | There was no measurable outcome with respect to maltreatment. | Severe psychopathology was determined in 36(60%) of the fathers. 31 (52%) fathers had major depression, 6 (10%) had schizophrenia.  11 of the 12 men who committed suicide after killing multiple children had a diagnosed mental health problem. 9 of these were suffering from major depressive disorder and 2 from psychosis. |  | | New born -35 years old. | N/a |
| Castro-Vale (2019) | Analysis of data obtained from the adult offspring of Portuguese war veterans with and without a PTSD diagnosis. Offspring completed a questionnaire and interview. | Cross sectional | 46 fathers suffering from PTSD and 15 controls.  46 and 15 adult children for these groups were sampled respectively. | Biologic | Current or previous PTSD diagnosis of the adult children’s father as assessed with structured clinical interview | Sexual Abuse  Physical Abuse Emotional Abuse  Emotional Neglect  Physical Neglect  Measured with the Child Trauma Questionnaire self-reported by children. | Analysis showed that a fathers’ lifetime diagnosis of PTSD had no significant predictive effect on emotional abuse (ß=0.2771, p=0.763), sexual abuse (ß=-0.383, p=0.564), physical abuse (ß=0.397, p=0.363), emotional neglect (ß=0.622, p=0.511) and physical neglect (ß=0.074, p=0.851). |  | | 21-44 | None |
| Čatipović (2003) | Investigated records of reported child abuse from Bjelovar Croatia. | Cross sectional | 91 children. 68 abusing fathers. | Both | Whether the father had had psychiatric treatment in the past as mentioned in the records. | There was no measurable outcome with respect to maltreatment. All children had suffered from maltreatment | In the 24 cases of emotional abuse, abusers were exclusively fathers, and none of these had been reported to have been treated for a psychiatric disorder. | In the analysed sample, female children were significantly more exposed to physical abuse (p = 0.040). There was no statistically significant difference in the relationship between gender and neglect (p = 0.465) or emotional abuse (p = 0.429). | | 1-17. | N/a |
| Clement (2016) | Telephone survey interviewing the parents of children in Quebec. Questions were asked concerning only one child per household. | Cross sectional | 1104 fathers 1104 children  20.6-29.4% of these children were neglected depending on their age | Both | Depressive symptoms (CES-D) | Emotional, cognitive, supervision and physical neglect as measured with the parent report multidimensional neglectful behavior scale. | Paternal depression symptoms caused a non-significant increase in the rates of neglect in parents with children aged 6 months-4 years (OR=1.5 CI=0.5, 4.3) or ages 5-9 (OR=3.1 CI=0.9, 9.9) or 10-15 years (OR=2.1 CI=0.9, 5.3). | Paternal stress relating to reconciling work with family significantly associated with increased levels of neglect in ages 6m-4y (OR=1.8 (1.0-3.2)) and 5 years to 9 years (OR=2.0 (1.0-3.9)) but not for children aged 10-15 (OR=1.6 (0.8-3.4)).  Depending on the child age category, rates of neglect varied between 20-26%. Emotional and cognitive neglect were the most common forms.  Perceived poverty caused significantly increased levels of neglect in the 6mo-4y group (OR=2.9 CI=1.1-7.5), and 10-15 year group (OR=3.1 CI=1.1-8.3). | | 6 months-15 years | Covariates included alcohol use, drug use, perceived economic situation and parental stress. |
| Coohey (2006) | Screened all reports of neglect in a county in a midwestern state between 1996-1998 and then tracked the records of each family for at least 2 years. | Retrospective Case control | 157 families who were deemed to have neglected a total of 378 children.  105 of these families had a biological or non-biological father present. | Both | If the CPS investigator had written in the reports that the mother’s partner either had a mental health problem, was undergoing therapy for a mental health problem or were taking medication for a mental health problem. | Compared the chronicity of neglect. The categories were: single time, persistent (2 or more substantiated cases of neglect for 2 years or less), or chronic (2 or more substantiated cases of neglect and involvement in CPS for 2 years or more). | The mother’s partner having a mental health problem was significantly different in families engaging in persistent cases of neglect compared to chronic cases (ß=2.96, AOR=19.38, p<0.05). The multinomial model showed no significance of the mother’s partner having a mental health problem when discerning characteristics of families in the isolated/chronic and isolated/ persistent categories. | Paternal problem with alcohol and drugs was significant in the multinomial logistic regression model comparing persistent/ isolated cases (ß=1.31, AOR=3.72, p<0.05) and chronic/ isolated cases (ß=1.66, AOR=5.28, p<0.05). | | No info | Covariates in the model were number of children, number of children below 6, whether mother had a partner, the number of parents living in the household, biological or non-biological parents, mother having problems with alcohol or drugs, maternal mental health problems, father having problems with mental health or drugs, mothers partner battered her and understanding of the problem. |
| Davis (2011) | Utilised the fragile families study data set. Performed cross sectional analysis of data collected at the follow up completed when the child was 12 months old. This only investigated fathers who lived with child almost all the time | Cross sectional | 1773 fathers  1773 children  41% of children whose fathers were diagnosed with depression were physically abused compared to 13% for who their fathers did not have such a diagnosis | Biologic | Depression diagnosis  (WHO composite international diagnostic interview followed by DSM-IV categorisation) | Parental reported physical abuse (Spanking) | Fathers with a diagnosis of depression had more than a 3x greater chance of engaging in physical abuse by spanking their children (OR=3.92 (CI=1.23, 12.5)). | 92% of non-depressed fathers were employed compared to only 69% of depressed fathers (p<0.01) | | 1 year old | Covariates which were controlled for included fathers age in years, race and ethnicity, household poverty level, education, employment status, paternal substance abuse and child gender. |
| Duffy (2015) | Investigated reports from families in the nurturing families network, Connecticut's child abuse and neglect program. Families in the program have been identified as having a higher risk of child abuse and neglect. The included families had had either a substantiated or unsubstantiated report made to the CPS. | Cross sectional | 131 families' reports were investigated. Of these, around 65 families had available data on paternal mental health. | Both | "Mental health history" of the father. This was based on CPS reports. | Whether the reports to the CPS which were made were substantiated or unsubstantiated. | Paternal mental health history caused a non-significant increase in chances that a claim would be substantiated (OR=2.22 (CI=0.56–8.77 )). | Paternal CPS history had no significant change in the chances of the investigation being substantiated (OR=0.88 (CI= 0.17–4.65)). | | 0-42 months. | Controlled for the gender of child, maternal age, race, language. |
| Eriksson (2016) | Utilised data from the Australian homicide project which interviewed perpetrators of murder and manslaughter offenses. Data was drawn to compare cases of filicide and non-filicide homicide. | Mixed qualitative and quantitative design | 231 Total. interviewed. 9 of these committed paternal filicides, 193 were male who committed non filicide homicides. | Both | Participants were asked if they have received treatment or assessment for a mental health issue in the year leading up to the homicide. | There was no measurable outcome with respect to maltreatment. | No fathers had treatment or assessment of a mental health issue in the year leading up to the filicide. 1 however from descriptions was suffering from MHD. 20.6% of the non-filicide males had treatment or assessment. |  | | 0-18 | N/a |
| Francis (2008) | Compared a group of Canadian fathers confirmed to have physically abused their children compared to a group of controls who had not. | Cross sectional | 24 confirmed physically abusive fathers and 28 non-abusive fathers.  24 abused and 28 non abused children. | Both | Symptoms of Depression  Anxiety  Psychoticism  Paranoia  (BSI) | Whether the father was physically abusive to the child | Abusive fathers had significantly higher rates of severe depression (50% vs 10% in clinical range, χ2=7.62, p<0.01).  There was a significant increase in the number of abusive fathers with a BSI anxiety score in a clinical range (47.6% vs 20.0%, χ2=3.48)  Psychotic symptoms were significantly higher in abusive fathers (42.9% vs 10%, χ2=5.93, p<0.05)  Paranoia symptoms were significantly higher in abusive fathers (55% vs 15.8%, χ2=11.90, p<0.001) | Abusive parents were significantly more likely to have experienced maltreatment when a child (86.24% vs 55.81%, F=20.28, p<0.01).  Abusive fathers’ highest educational grade level was significantly lower than that of non-abusive fathers (p<0.001) .  Significantly fewer parents in the abusive group had annual household income above $39,999 (9.5% vs 60.0%, χ2=12.48,p<0.001) and significantly more had annual household income below $20,000 (52.4% vs 16.0%,χ2=6.87, p<0.01). | | Under the age of 12 | Comparison groups did not differ in age, race or employment status. Education was used as a covariate. |
| Fredman (2019) | Investigated mother-father dyads who were taking part in a home visiting program in Ohio and Southwest Kentucky. Mothers needed to have one of the following for eligibility: unmarried, low income, <18 years, inadequate prenatal care. Mothers and fathers had to be between 18-50, speak English and agree they were committed to raising the child together. | Cross sectional | 150 dyads | Biological | PTSD symptom severity (assessed with PTSD symptom checklist) | Potential of father to commit physical child abuse (CAPI) | Father's PTSD correlated with father's potential to commit child abuse (correlation=0.53, P<0.001), but not that of Mother’s (correlation=0.03, ns).  There was a significant direct actor effect from PTSD symptom severity to child abuse potential in both fathers (ß=0.51, p<0.001) and mothers (ß=0.51, p<0.001).  There was a significant effect of the interactions between mothers’ and fathers’ PTSD symptoms in predicting fathers’ child abuse potential (ß=0.15, p=0.031). | Fathers’ PTSD was significantly correlated with fathers’ conflict (correlation= 0.25 P<0.001) and fathers love (correlation= -0.18 P<0.01) and mothers’ conflict (correlation=0.18 P<0.001). | | Conducted interviews at either the 5th month of pregnancy or three months postpartum. | Covariates included mother and father’s age, cohabitation status, prenatal vs postnatal status, years of education and father’s work status and incarceration history. |
| Friedman (2005) | Utilised families’ files from the Cuyahoga county coroner's office (Cleveland) in cases of filicide-suicide which occurred between 1958-2002. | Cross sectional | 20 fathers who committed filicide | Biological | If the father had a mental health disorder as per the coroner’s reports. | There was no measurable outcome with respect to maltreatment. | 15 fathers suffered from a mental health disorder (75%). 5 had psychosis, 10 had depression and 4 had delusional disorder. |  | | 3-17. Mean age of 7.1 | N/a |
| Garosi (2021) | Utilised data from FACE-SZ cohort. All participants had schizophrenia or schizoaffective disorder. Asked participants to self-report their parents' diagnosis and own experience. | Retrospective case control | 724 adult children reporting on their childhood | Biological | History of schizophrenia or bipolar disorder | Physical abuse, emotional abuse, physical neglect, emotional neglect, sexual abuse (CTQ score) | Multivariate analysis reported children whose father had schizophrenia or bipolar disorder had increased mean physical abuse (aOR=0.081, p=0.042) and emotional abuse mean scores (aOR=0.146, p<0.001). | Fathers with history of schizophrenia or bipolar disorder had increased rates of current major depressive disorder (aOR=2.903, p=0.009), lifetime number of psychiatric hospitalisations (aOR=0.160, p<0.001) and longer psychiatric hospitalization duration (aOR=0.088, p=0.041). | | Reports of an adult on their childhood | Covaiates included age, sex, education level, having children, being with a partner, employment, living in own home, illness characteristic, lifetime number of psychiatric hospitalisations, current major depressive disorder. |
| Green (2021) | Cohort study of Australian children from pre birth to age of 13-14 | Cohort | 72,059 Children | Biological | Any “Parental mental disorder” (ICD-110) | Substantiated CPS reports or placement in OOHC before age 13-14 | Parental mental disorder predicted child maltreatment (OR= 2.175 (99%% CI = 1.77 - 2.65). | Childhood maltreatment also related to mother younger than 21 years (OR= 2.80, CI=2.47, 3.18), no/late antenatal visit (OR=1.30, CI= 1.17, 1.44), maternal smoking during pregnancy (OR=2.71, CI=2.43, 3.03), preterm birth (OR=1.45, CI=1.22, 1.71), >2 previous pregnancies (OR=2.57, CI=2,25, 2.93), prenatal CPS report (OR=8.18, CI=5.29, 12.73), maternal offending (OR=3.03, CI=2.67, 3.45), paternal offending (OR=2.57, CI=2.31, 2.86) and maternal mental disorder (OR=3.54, CI=3.08, 4.07). | | 0-14 | Coviates included child sex, mother being under 21, pregnancy complication, no/late antenatal visit, maternal smoking during pregnancy, socioeconomic disadvantage, prenatal CPS report, maternal and paternal offending and maternal and paternal mental disorder |
| Haapasalo (2000) | Investigated the records of imprisoned Finish young offenders. Data was obtained by utilising the CPS, social services, clinic and hospital records of these individuals. | Cross sectional | 78 young offenders whose CPS records could be recovered. | Information not found | Any clear mention of a mental health condition of the father in the CPS/ social worker/ hospital files of these individuals. | Substantiated reports of:  Physical abuse  Psychological abuse  Neglect  (All as written in the case files).  Numbers of CPS interventions. | A mention of a paternal mental health problem was significantly correlated with the number of CPS interventions (correlation=0.27, p<0.05). A paternal mental health problem was not significantly correlated to physical (correlation= 0.00, p>0.05), psychological (correlation= 0.17, p>0.05) abuse and neglect (correlation= 0.09 p>0.05).  A paternal mental health problem resulted in a non-significant increase in the number of CPS interventions (ß=0.11, p>0.05). | A paternal mental health problem was significantly correlated with paternal alcoholism (correlation=0.28, p<0.05)  Financial problems of families significantly correlated with the number of CPS interventions (correlation=0.35, p<0.01). It however resulted in a non-significant increase in the number of CPS interventions when analysed utilising the multivariate model (ß=0.10, p>0.05). | | No info | Covariates were spousal, physical and psychological abuse, maternal mental health, paternal and maternal conviction for a crime, maternal and paternal alcoholism, financial problems, neglect. |
| Harris (2007) | Analysed police reports of all filicide cases in Canada which were entered into a national database between 1996-2003. | Cross sectional | 86 children killed by biological fathers, 62 by non-biological fathers | Both | Serious mental problems score- calculated by counting the number of distinct terms (e.g., depression, psychosis, delusion, hallucination and extreme stress) used to describe the perpetrator. | There was no measurable outcome with respect to maltreatment. | Biological fathers had a statistically greater mean score (0.43 (+-0.16) compared to non-biological fathers (0.27 (+-0.15)). |  | |  | N/a |
| Heckler (2022) | Investigated risk factors for child-directed parental violence in Burundin families living in refugee camps in Tanzania. | Cross-sectional | 226 fathers and children | Both | PTSD symptom severity (PTSD checklist for DSM-5), Depression symptoms (BSI-18). | Physical abuse and emotional abuse reported by child (Parent-child conflict tactics scale) | PTSD symptoms were associated with a non-significant increase in physical and emotional abuse.  Depressive symptoms were associated with decreased physical and emotional abuse (ß=0.19, p<0.05) | Father’s emotional and physical abuse was predicted by education (ß=-0.10, p<0.05) and being a biological father (ß=0.12, p<0.01). | | 7-15 | Covariates of the father and mother characteristic included education, being a biological father, childhood experience of violence, alcohol use and psychosocial dysfunction.  Covariates of the child included age, gender, externalising symptoms and PTSD symptoms |
| Jakupčević (2011) | Utilised data from interviews and questionnaires from outpatients with a psychiatric diagnosis of PTSD compared to both controls and outpatients with anxiety. This study was conducted in Split. | Cross sectional | 30 men with a diagnosis of PTSD. 100 controls (45 fathers, 55 mothers) and 50 anxiety suffers (25 men 25 women) | Both | Psychiatric diagnosis of PTSD.  Psychiatric diagnosis of mixed anxiety and depression disorder (MADD). | Potential of father to commit physical child abuse (CAPI) | There was greater risk of child abuse (indicated by a higher CAPI score) in fathers with PTSD (333.2+-18.0) compared to parents from the general population (76.9+-9.9).  Fathers with PTSD had a higher potential for child abuse than parents with MADD (t=13.73, P<0.001). | Parents with MADD or PTSD reported more experiences of childhood abuse (F=14.46, P < 0.001).  Including sociodemographic variables included in the model explained 40% of the variance of the risk of physical abuse of children, with age, educational status, and financial status being significant. | | 5-11 |  |
| Kaplan (2009) | Investigated families in 2 counties in New York state who were referred to CPS for physically abusing their children compared to control families who had not. | Cross sectional | 99 children abused and 99 controls.  (Data was available from 52 and 70 of these fathers respectively) | Biological | Lifetime and current psychiatric disorders of parents as measured by SCID I for DSM III R.  Beck depression inventory for measuring severity of depression. | Physical abuse | Fathers of referred children had an increased prevalence of any depressive disorder in their lifetime (34.6% vs 14.3%, p<0.01). Fathers of referred children had increased prevalence of any anxiety disorder in their lifetime (19.2% vs 2.9% p=0.0029).  Logistical regression analysis was performed. Abuse group membership was significant over and above covariates for lifetime paternal anxiety disorder (χ2=6.59, df=1, p <0.01). Lifetime paternal depressive disorder was not significant over and above other covariates. | In multivariate logistic regression analysis, paternal substance/ alcohol abuse disorder was highly significant as a predictive factor (OR=5.73 (CI=1.61,20.33), p=0.0070).  Abuse group membership was significant over and above covariates for maternal depressive disorder (χ2=8.10, df=1, p <0.01). | | 12-18 | Child age, gender, community income were matched for controls.  Educational achievement, parental age and marital status were all entered into the regression model. |
| Kauppi (2010) | Investigated cases of filicide in Finland occurring between 1970-1994. Data was obtained from statistics Finland which kept records. | Cross sectional | 20 cases of paternal non neonaticide, non-filicide-suicide filicide. | Both | Diagnosis made after the murder by clinicians. Based on ICD-9 and DSM-III. If this wasn’t required in the case then a previous psychiatric diagnosis was used. | There was no measurable outcome with respect to maltreatment. | Of the 15 that underwent assessment, 2 had psychosis, 1 had psychosis and substance abuse disorder, 1 had depression and substance abuse disorder/ personality disorder, 4 had personality disorder, 5 had personality disorder and substance abuse disorder and 2 had personality disorder features. |  | | 1-14 | N/a |
| Kelley (2015) | Investigated a group of heterosexual couples from New York or Virginia, where at least one meets the DSM-IV criteria for drug or alcohol abuse. | Cross sectional | 85 couples.  52 only fathers had SUD, 33 both parents did. | Both | Depressive symptoms were measured by self-report of the father filling in the CES-D. | Potential of father to commit physical child abuse (BCAP).  Over reactivity as per parenting scale. | Paternal depressive symptoms significantly predicted an increased potential of the father to commit physical abuse (ß=0.38, p<0.001). | Maternal depressive symptoms significantly increased the chance of the mother to commit physical abuse (ß=0.38, (p<0.001).  Paternal depressive symptoms were not significantly predictive of the potential for maternal abuse. | | Below 18. |  |
| Lee et al (2011) A | Utilised the fragile families survey. Investigated Hispanic fathers who were residing in the home at the times of the follow up interviews when the child was 3 and 5 years old. Compared fathers who were and were not native to the United States. | Cohort | 372 fathers  372 children  18 children suffered from physical and 48 from psychiatric aggression | Biologic | Depression diagnosis as per DSM-III-R criteria. | Physiological and physical aggression as assessed by the CTSPC reported by the mother. | The father having a major depression diagnosis when a child was 3 did not predict physical (ß=0.02, p>0.05) or psychological (ß=0.23, p>0.05) aggression when the child was 5. | Nativity status of the father (being foreign born) predicted use of physical (ß=0.25, p<0.01) and psychological (ß=0.24, p<0.05) aggression when the child was 5 years old.  In a path model, controlling for other covariates, paternal major depression at the 3 year interview resulted in a non-significant mediating effects of nativity status of father on physical (ß=0.02) and psychological (ß=0.23) aggression at 5 years.  Heavy alcohol use at 3 years significantly mediated the association between nativity status and physical aggression at 5 years (ß=0.31, p<0.05). | | Child aged 3-5 | Covariates included alcohol use, parenting stress, and involvement in caregiving, IPV, gender of child, paternal age, household income  and controlled for the child’s aggressive behavior. |
| Lee et al (2011) B | Utilised the fragile families survey. Data was collected from the interviews at baseline at birth and assessment at the follow up when the child was 3 years old. | Cross sectional | 2309 children and their fathers.  901 reported the use of corporal punishment. | Biological | Major depressive disorder or anxiety diagnosis as per CIDI-SF at baseline. | Physical abuse- corporal punishment (CP) frequency reported at the 3 year follow up. Reports were categorised into no corporal punishment, moderate corporal punishment and heavy corporal punishment depending on the frequency of corporal punishment occurring in the last month. | The presence of major depressive disorder increased the odds of heavy (OR=1.53 (CI=1.06,2.21), p<0.05), but not moderate (OR=1.31 CI=0.93-1.84) CP.  GAD did not significantly increase the incidence of moderate (OR=0.78 (CI=0.39-1.55)) or heavy (OR=0.71 (0.32-1.58)) CP. | Hispanic families have lower occurrence of heavy CP (OR= 0.68 (CI=0.46-0.99)).  African American families have higher levels of moderate CP (OR=1.63 1.19-2.23 p<0.01).  Failed to show a significant association between household income and incidence of moderate (OR=1.06 (CI=0.97-1.16)) and heavy (OR=1.03 (CI=0.93-1.13)) CP.  Fathers who were not married or cohabiting have much lower levels of moderate (OR=0.41 (CI=0.30-0.56), p<0.001) and heavy (OR=0.26 (CI=0.18,0.38), p<0.01) CP. | | All aged 3 | Controlled for paternal involvement with the child and a range of sociodemographic variables linked to child maltreatment. Covariates included race, ethnicity, age at childbirth, marital status, education, a range of child characteristics, alcohol use and household income. |
| Lee (2013) | Utilised the fragile families survey. Analysed data from fathers who had been present at both the 3 and 5 year follow ups. | Cohort | 1089  1089 children  126 children were neglected, 64 had CPS involvement. | Biological | Depression diagnosis of father (assessed with CIDI-SF: section A) at the 3 year visit. | Physical and supervisory neglect (CTS-PC).  Referral to child protection.  Both were assessed at the 5-year visit. | A fathers’ diagnosis of depression at 3 years more than doubled the risk of neglect at 5 years (OR= 2.04 (CI=1.22, 3.42), P<0.01))  A paternal depression diagnosis at 3 years significantly increased the rate of CPS service involvement at 5 years (OR=2.42 (1.05, 5.58, P<0.05). | Hispanic families showed a significant increase in neglect in the logistic regression model (OR= 1.58 (CI=1.03–2.42), p<0.05.)  The current employment status of the father was significantly associated with neglect (OR=2.28 (1.36–3.84), P<0.01).  Government financial assistance was significantly associated with CPS involvement (OR= 2.18 (1.09–4.37), p<0.05). | | Child aged 3-5 | Covariates included household economic hardship, race, parental relationship quality, male child, child health, low birth weight, father’s age and education, parenting stress. |
| Marleau (1999) | Utilised data from psychiatric, psychological and criminological assessments found in the files of filicidal men in a Canadian inpatient facility between 1982-1994. | Cross sectional | 10 fathers who had each committed one act of filicide | Both | DSM-III-R classification of each man occurred shortly after clinical interview. | There was no measurable outcome with respect to maltreatment. | All 10 were diagnosed with either an axis I or axis II disorder. 4 had a mood disorders on axis I. 8 had personality disorders on axis II. 4 were psychotic at the time of the offense. 1 had schizophrenia. |  | | 9 months - 14 years | N/a |
| Putkonen (2010) | Investigated records of all filicide offenders in Austria and Finland between 1995-2005. Data was gleaned from statistics Finland and the Coroner Institutions of Austria. | Cross sectional | 45 fathers.  Between 45-50 children were killed. | Both | Diagnosis made after the murder by clinicians. Based on clinical interview utilising ICD-9 and DSM-III criteria. | There was no measurable outcome with respect to maltreatment. | Prior to the act of filicide, no father had received an official diagnosis. Post filicide, 95% of fathers received a diagnosis as per DSM-III criteria. 13% of fathers were diagnosed with psychotic disorder, 30% with non-psychotic depression, 57% with personality disorder. |  | | No information | N/a |
| Rodriguez (2018) | Utilised the following families study which followed families in the south east of the United States. Parents were assessed in the last trimester of pregnancy and when the child was 6 and 18 months old. | Cohort | 203 mothers (151 partners available and were included) | Both | Self-report of depression and anxiety by father filling in the BSI at all times that the parents were assessed. | Parent child aggression (PCA) risk as a composite score of CAPI, AAPI form B and ReACCT. | A change in a father’s psychopathology symptoms over time significantly predicted a change in PCA risk for that father (ß=0.239 (CI=0.019,0.447), p=0.038). If there was an increase or decrease in a father’s psychopathology it predicted an increase or decrease in PCA risk respectively.  The cohort was separated into an at socioeconomic high risk group based on 2 of: public assistance, being 150% below the poverty line, high school education or less, 18 or younger. The effects of the fathers’ psychopathology was stronger for fathers in the risk group compared to the low risk group (β= −0.239 (CI=0.019, 0.447), p= .038). | There was a significant association with PCA approval, negative attritions, emotional regulation, and couple satisfaction.  There was no significant effect of knowing other discipline alternatives, SUD, IPV, social support satisfaction. | | Risk at pre term, 6 months and 18 months | Covariates in the model included PCA approval attitudes, knowing discipline alternatives, negative attributions, substance abuse issues, IPV, emotional regulation, couple satisfactions and social support satisfaction. |
| Sachs-Ericsson (2012) | Used results from the NCS-R (National comorbidity survey- representation). This represented a nationally representative sample of Americans who received a psychiatric interview and completed a subsequent survey. | Retrospective Case control | 2960 adult children reporting on maltreatment and parental mental health.  889 of these children suffered from maltreatment. | Information not found. | Asked to report on symptoms of fathers internalising and externalising disorders using the family history research diagnostic criteria interview. | Physical Abuse  Sexual abuse  Self-reported by adults in their childhood before the age of 15. | A significant causal relationship was found between a fathers’ externalising symptoms and any form of abuse (ß = 0.245, p<0.001). A significant causal relationship was not found between fathers’ internalising disorder symptoms and any form of abuse (ß=0.063, p=0.071). | Maternal internalising disorder symptoms had a causal relation to abuse (ß=0.081, p=0.010).  Mothers' externalizing symptoms had a non-significant causal relationship to any form of abuse (ß=0.058, p=0.106). | | Mean age 44.9 | Covariates included early parental loss or separation, reliance on welfare, sex, education, income and age. |
| Scharpf (2020) | Analysis of structured interviews performed with families living in a refugee camp in Western Tanzania. | Cross sectional | 226 children and 226 fathers. | Both | Parent psychological distress measured by the BSI-18 and PTSD symptoms measured utilising the PTSD checklist for DSM-5. | Physical abuse  Psychological abuse, neglect  (Maltreatment measured by Parent-Child Conflict Tactics scale) | Paternal psychological distress caused a non-significant increase in rates of child maltreatment (ß=0.09, SE=0.09, p>0.05).  Paternal PTSD symptoms had a non-significant correlation with maltreatment by parents (correlation=0.08, p>0.05). | Paternal psychopathology had a direct effect on child psychopathology (ß=0.17, p<0.5).  Maternal psychopathology caused a significant increase in rates of child maltreatment (ß=0.20, p<0.01). | | Aged 7-15 | Attachment representations of parents, child age and sex, child PTS symptoms, child emotional and behavior problems. |
| Schaeffer (2005) | Analysed questionnaire results from parents at 27 Army installations in US, Germany and Japan who were receiving services through the Army New Parent support program. All parents were receiving home visitation services at the time of data collection. | Cross sectional | 590 mothers and 175 fathers  16% of families had a case of child maltreatment substantiated by local CPS.  22% of fathers were considered to be at high risk for child maltreatment. | Both- 82% of the fathers were biological | Depressive symptoms- measured by self-report of father filling in the CES-D. | Potential of father to commit physical child abuse (CAPI) | Depressive symptoms of fathers (ß=0.55, t=10.33, p<0.0001) were significantly related to an increased score on the CAPI indicating greater potential to commit child abuse. | Father’s stress (ß=0.15, t=2.78, p<0.01), family conflict (ß=0.14, t=2.28 p<0.05, and family expressiveness (ß=-0.10, t=-1.99, p<0.05) as measured by FES were also significantly related to an increased score on the CAPI. Poor marital adjustment, dissatisfaction with social support networks, and low family cohesion predicted child abuse potential for mothers but not for fathers.  Depressive symptoms of mothers (ß=0.57, t=20.23, p<0.0001) were significantly related to an increased score on the CAPI. | | 0-6 | Controlled for parent age, education level and race. |
| Sidebotham (2001) | Analysed data from the Avon study. This study sent 4 questionnaires to mothers during pregnancy.  The local social services’ child protection registers were also used. Searched for any children with birth dates that matched the range of the Avon study. | Nested case control | 14138 children,  162 of which were identified as being maltreated  13976 fathers | Both | A positive paternal history of depression or "other psychiatric illness". This was reported by the parents during pregnancy. | The child having later been on the child protection services register for suffering from (or being at high risk of) physical, emotional abuse or neglect | After controlling for other variables, paternal depression largely predicted being on the CPS register (OR=3.6 (CI= 1.63, 7.96), P<0.005).  Paternal psychiatric illness excluding depression was not significantly correlated with a child being on the CPS register (OR= 1.52 (CI=0.31, 7.54), p=0.61). | In the logistic regression model, being on the CPS register was significantly predicted by paternal age under 20 (OR=6.33 (CI=2.76,14.51) ,P<0.001) and fathers highest educational level being GCSE/ vocational training (OR=3.58 (CI= 1.66,7.73), p<0.005).  Maternal depression was not significantly predictive for maltreatment in multivariate analysis. | | 0-6 | Covariates entered into the regression model included maternal age at delivery, parent’s highest educational qualification, if parents were sexually abused, parental bonding index: overprotection and maternal care, alcoholism and drug addiction. |
| Stearns (2019) | Students at a southern United States university retrospectively reported on their parent’s mental health and their experiences of maltreatment.. | Retrospective case controls | 350 male and 662 female students. | Both | Parental depression and anxiety symptoms as measured by the adult behaviour checklist. | Physical abuse  Psychological abuse (CTSPC) | Psychological maltreatment was significantly directly affected by paternal depressive problems in daughters (ß=0.22 p<0.05), and paternal anxiety problems in sons (ß=0.19, p<0.05).  Physical maltreatment was significantly affected by paternal depression problems in sons (ß=0.26, p<0.05) and daughters (ß=0.32, p<0.05) but there was no significance for anxiety. | Maternal anxiety problems had a direct effect on psychological maltreatment in both sons (ß=0.17, p<0.05) and daughters (ß=0.17, p<0.05).  Maternal depressive problems had a direct effect on physical maltreatment in both sons (ß=0.10, p<0.05) and daughters (ß=0.23, p<0.05). | | Children were aged between 18-25 at the time of reporting | Irritability and defiance were included in the path analysis. |
| Takehara (2017) | Recruited Japanese families who completed a questionnaire at around 8-12 weeks’ gestation. This data was analysed along with follow up questionnaires received in the first few days, one month and two months postpartum. | Cohort | 196 fathers  196 children | Both | Depression diagnosis as per the Edinburgh scale. | Maltreatment practices as per the child maltreatment scale assessed at 2 months postpartum. | The odds of child maltreatment tendency did not significantly differ between fathers with prenatal depression and those without it (OR= 1.62 (CI= 0.41, 6.44)) or between fathers with prior paternal depression after birth (between birth and 2 months) and those without it (OR= 3.67 (CI= 0.97, 13.66). However, participants with current paternal depression (i.e., at 2 months postpartum) had a significantly greater OR of having child maltreatment tendency at this time (OR= 7.77 (CI= 1.83, 33.02)). | There was a significant increase in the incidence of maltreatment when parents had more than one child (28.8% vs 4.9%, p<0.001). | | 2 months old | Adjusted for paternal age, education, employment status, family income, wanted pregnancy, parity, history of paternal depression, maternal depression at two months postpartum and low birth weight. |
| Walsh (2002) | Utilised data from the Ontario Health Supplement which was generated from interviews of the general population of Ontario (aged above 15). These subjects reported on the mental health of their parents and their experiences of maltreatment. | Retrospective case control | 8548 adults self-reporting  2453 reported either sexual or physical abuse | No information was found. | Asked if their parents suffered from, or exhibited symptoms of depression, manic depression, schizophrenia and antisocial behavior. | Physical Abuse  Sexual abuse | Father suffering from any form of mental health disorder resulted in increased chances for physical (OR=2.4 (CI=1.6, 3.4)) or sexual (OR=2.3 (CI=1.4, 3.6)) abuse. | Both parents suffering from any psychiatric disorder resulted in increased rates of reporting physical (OR=3.8 (CI=2.5, 5.8)) and sexual (OR=5.6 (CI=2.8, 11.2)) abuse.  Mothers suffering from any form of mental health disorder resulted in increased chances for physical (OR=2.5 (CI=1.9, 3.2)) or sexual (OR=3.8 (CI=2.7, 5.4)) abuse. | | Reports of an adult on the whole of their childhood | Regression estimates were controlled and adjusted for gender, parents’ education, low income and age of respondent. |
| Whitten (2020) | Utilised data from the new south wales child development study which linked data from multiple government agencies (e.g., healthy, education, justice and CPS) for children between the ages of 0-14. | Cohort | 71,661 children, 71532 fathers  16,585 children had contact with CPS  1,287 children had contact with out of home care (OOHC) | Biological | Diagnosis of an ICD-10 AM mental health disorder before first contact with social services. | Chance of child protection contact.  Chance of placement in OOHC. | Results from univariate analysis indicated that prior paternal mental health diagnosis had a significant effect on the incidence of earlier child protection contact (HR=1.67 (CI=1.55, 1.29)). A prior paternal mental health disorder had no association with OOHC placement (HR= 0.84 (CI= 0.68, 1.04)).  Results of multivariate analysis after controlling for cofactors indicted that a prior paternal mental health disorder had significant effects on any chance of child protection contact (HR=1.05 (CI=1.00, 1.10)) but not to OOHC contact HR=1.06 (0.94, 1.19). | In multivariate analysis, socioeconomic disadvantage significantly predicted any child protection contact (HR= 1.17 (CI= 1.13, 1.21)).  Individuals with prior paternal criminal conviction) had almost a two-and-a-half times greater incidence of first child protection contact (HR=2.27 (CI= 2.14, 2.40)).  Torres strait islander status showed large relation to any child protection contact (HR=5.99 (CI=5.64,6.36)) and OOHC placement (HR=3.05 (CI=2.72-3.41)). | | 0-14 | Covariates included mothers age at birth, Aboriginal or Torres Strait islander status, prenatal maternal smoking, relative socioeconomic disadvantage and maternal or paternal conviction. |
| Witte (2018) | Analysed data from an online survey completed by 2 siblings recruited online via online advertisement, university mailing lists and the “SoSci panel”. Available to German speaking individuals over the age of 18. Participants reported on their experiences of parental mental ill health and child abuse. | Retrospective case control | 870 sibling pairs self-reporting  404 pairs both reported maltreatment  163 only the older sibling reported maltreatment  125 only the younger sibling reported maltreatment | Both | Whether a participant mentioned that any of their family members had “psychological problems” including alcohol and drug problems. | Sexual Abuse  Physical Abuse  Emotional Abuse  Emotional Neglect  Physical Neglect  (Assessed by the childhood trauma questionnaire) | Reports of the father having a mental health problem when reported by both siblings significantly increased the incidence of physical (OR=3.96 (CI=1.12, 14.02),p<0.05), and emotional (OR=4.38 (1.93, 9.93), p<0.001) abuse and physical (OR=4.92 (2.24, 10.78), p<0.001) and emotional (OR=3.79 (1.75, 8.20), p<0.01) neglect being reported by both siblings.  A paternal mental health disorder significantly increased the incidence of reporting every type of maltreatment of solely the younger sibling. This effect was not seen for the older sibling: physical abuse, sexual abuse, physical and emotional neglect did not show a significant increase. | Reports of the mother having a mental health problem significantly increased the risk of every type of maltreatment for solely the older sibling. | | Reports of an adult on the whole of their childhood |  |
